# A case of mitral regurgitation due to bilateral undifferentiated papillary muscles with detailed preoperative diagnosis of associated morphological abnormalities

**DOI:** 10.1186/s44215-024-00152-8

**Published:** 2024-05-06

**Authors:** Masato Hayama, Chihaya Ito, Yuichi Morita, Masayuki Shimizu, Masato Furui, Hitoshi Matsumura, Yoshio Hayashida, Mizuki Sumi, Go Kuwahara, Kiyoyuki Eishi, Hideichi Wada

**Affiliations:** 1 Department of Cardiovascular Surgery, Fukuoka University Faculty of Medicine, Nanakuma 7-45-1, Jyonanku, Fukuoka-Shi, Fukuoka 814-0180 Japan; 2 Department of Cardiovascular Surgery, Hakujyuji Hospital, Kamisu, Japan

**Keywords:** Mitral valve plasty, Mitral regurgitation, Undifferentiated papillary muscles

## Abstract

Incomplete differentiation of the mitral valve structures during fetal life results in the papillary muscles and tendon cords becoming hypoplastic, a condition termed undifferentiated papillary muscles (UDPM). This fetal abnormality causes a group of diseases that cause mitral valve dysfunction in adult life. Here, we report a case of UDPM centered on the medial posterior apex in which detailed morphological abnormalities were diagnosed preoperatively using echocardiography and three-dimensional computed tomography (3DCT) analysis, contributing to plastic surgery.

The patient was a 64-year-old man who was followed up for several years for atrial functional mitral regurgitation (MR), ultimately developing heart failure. His MR was severe, and he was referred for surgery.

Echocardiography revealed restrictive tendon cords primarily in the posterior apex, abnormal muscle bundles in the lower part of the valve, and findings indicative of UDPM; this was considered the main cause of MR. The patient had surgery findings similar to the preoperative diagnosis, which greatly aided the surgery. Postoperatively, the MR was well-controlled and mild.

UDPM may present as various morphologic and functional abnormalities, including partial club-shaped tendinous MR and rheumatoid mitral stenosis, which are rarely accurately diagnosed preoperatively. Detailed analysis of morphological abnormalities using 3DCT or other methods may be beneficial for a successful plasty.

## Introduction

Undifferentiated papillary muscles (UDPM) is a condition rarely diagnosed preoperatively. The morphology of the abnormal subvalvular tissue varies among cases, and cases with a detailed evaluation of the area of abnormal mitral valve function have yet to be reported.

In this report, we describe a case of UDPM centered on the medial posterior apex, in which detailed morphological abnormalities were diagnosed preoperatively using echocardiogram and three-dimensional computed tomography (3DCT) analysis, contributing to plastic surgery.

## Case report

**Fig. 1:**
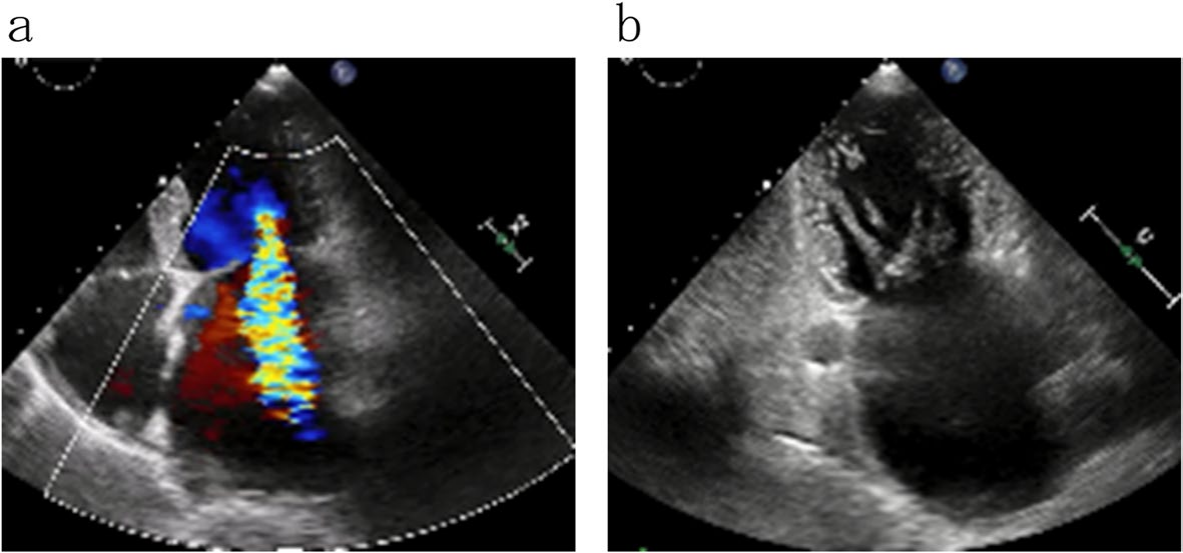
**a** Mobility of the posterior apex is reduced, and the regurgitation is directed toward the apex side, indicating tethering. **b** Normal papillary muscles and tendon cords cannot be observed beneath the valve. Thickened tendon cords appear to be fused and attached

A 64-year-old male who was undergoing follow-up for chronic atrial fibrillation was admitted to the hospital because of heart failure, whereupon echocardiography revealed severe mitral regurgitation (MR). On echocardiography, the MR flow was tethering-like and apex-lateral (Fig. 1a). Normal papillary muscles and tendon cords were not observed in the subvalvular tissue, and the tendon cords were fused (Fig. 1b), leading to a suspicion of MR due to UDPM. Additional cardiac CT scans were performed to examine the detailed morphology of the abnormal structures in the subvalvular tissue.

**Fig. 2:**
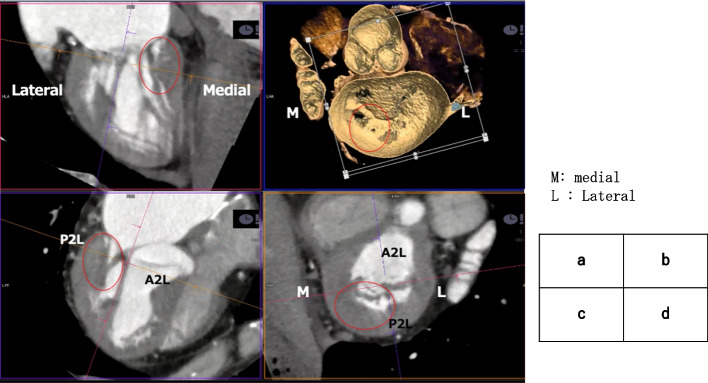
**a**, **b** Most of the tendon cords of the anterior and posterior apex are thick and fused. The medial side is directly attached to the posterior wall of the left ventricle, which seems to reduce the mobility of the posterior apex. **c**, **d** On the lateral side, which appears to be well-connected, P2 lateral is also directly attached to the left ventricle, resulting in decreased mobility

**Fig. 3:**
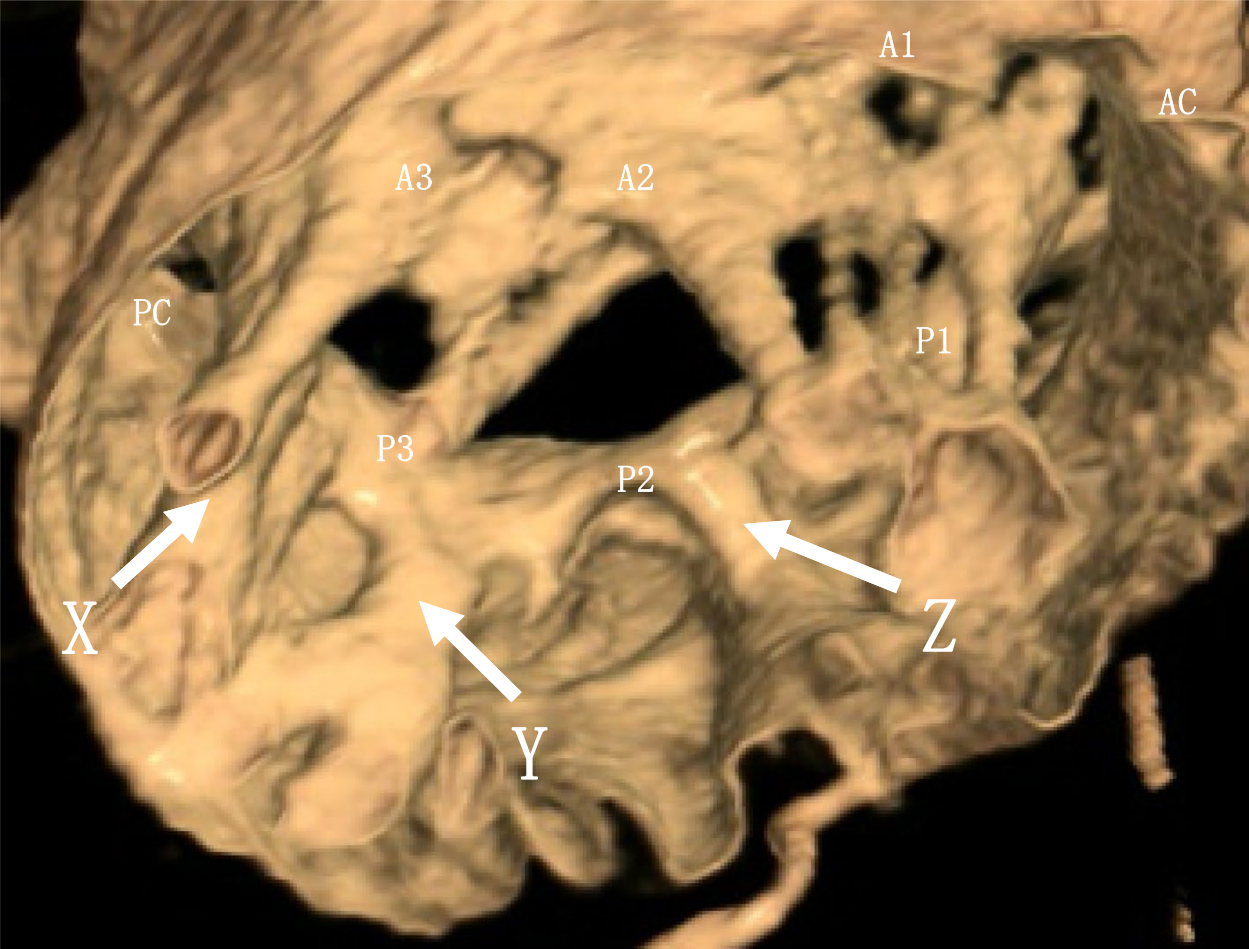
*X* is directly attached to the posterior wall of left ventricle on the medial side, and *Y* restricts the movement of posterior apex. *Z* is identified as part of the P2 lateral that has reduced mobility. The anterior apex is also undifferentiated but still mobile

Cardiac CT tomography revealed that most of the tendon cords of the anterior and posterior apices were thick and fused. The medial side was directly attached to the posterior wall of the left ventricle, which seemed to reduce the mobility of the posterior apex (Fig. 2a, b). On the lateral side, which appeared to be well connected, the P2 lateral was also directly attached to the left ventricle, resulting in decreased mobility (Fig. 2c, d). In the overall view, *X* was directly attached to the posterior wall of the left ventricle on the medial side, and *Y* restricted the movement of posterior apex. *Z* is part of the P2 lateral with reduced mobility. The anterior apex was undifferentiated but mobile (Fig. 3).

**Fig. 4:**
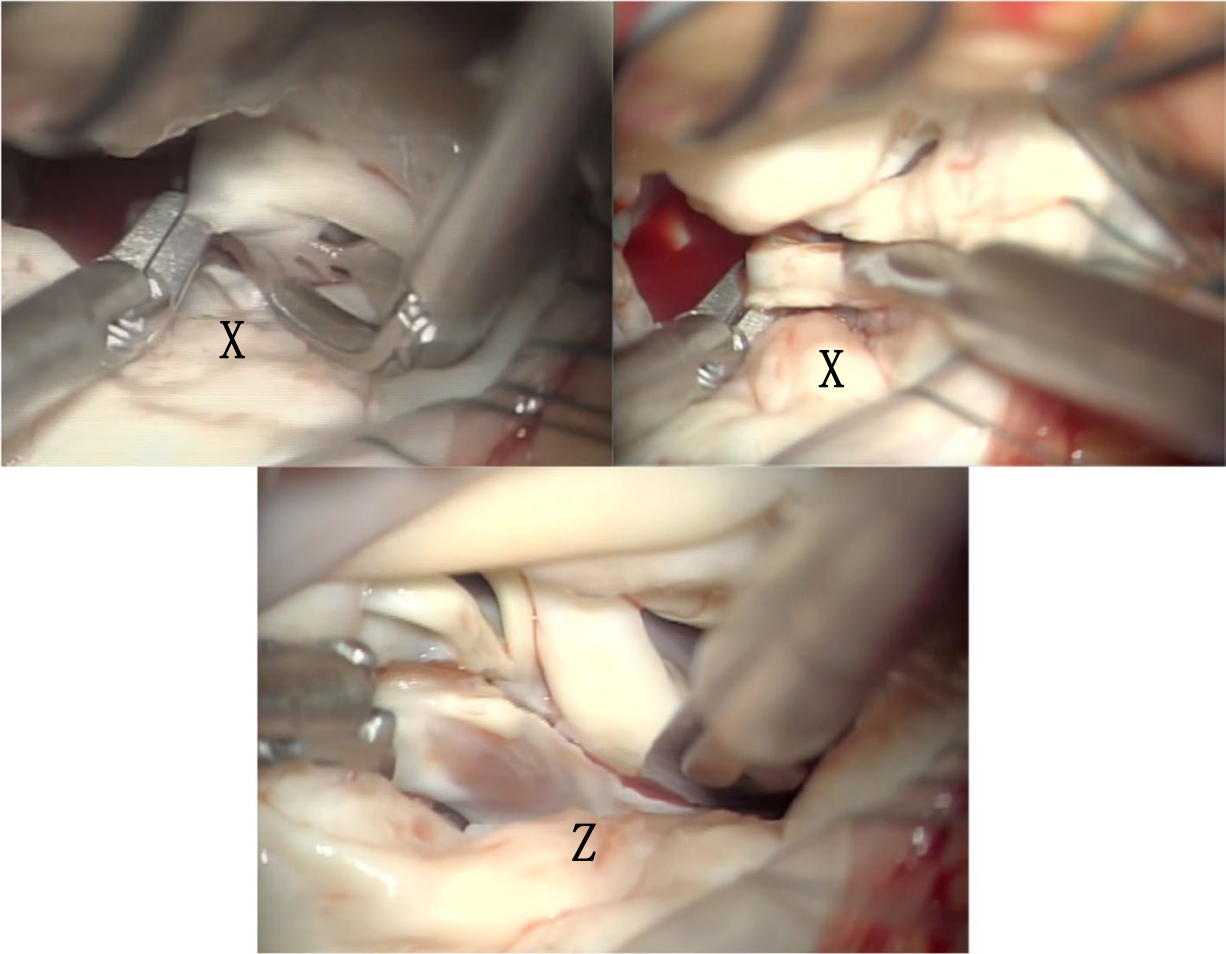
The *X* and *Z* tendon cords, which were directly attached to the myocardium and had reduced mobility, were resected and some were sliced to improve the mobility of the valve leaflet

**Fig. 5:**
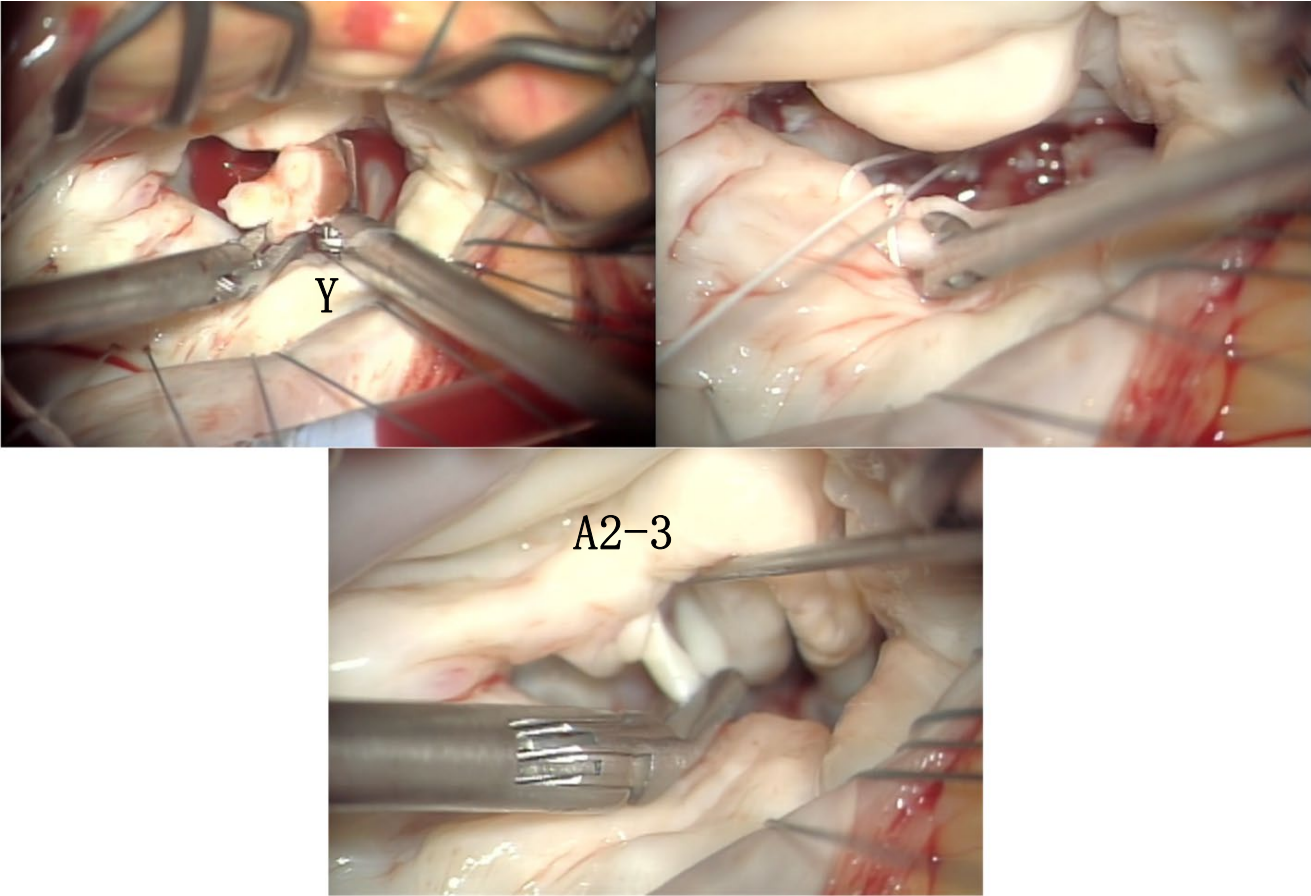
The *Y* tendon cord was resected and an artificial tendon cord was erected using CV-4. The tendon cords of the anterior apex were also undifferentiated

Surgery comprising median thoracotomy was performed. A progressive myocardial protection catheter was inserted into the ascending aorta, and aortic cross-clamping was performed. The mitral valve was approached through a right lateral left atrial incision. An atrial lift retractor was used to open the left atrium and expose the mitral valve. The subvalvular tissue was similar to that observed on the preoperative cardiac CT scan. The tendon cords of the anterior apex were undifferentiated, but as the mobility was good as seen in the CT findings, no treatment was performed. The *X* and *Z* tendon cords, which were directly attached to the myocardium and had reduced mobility, were resected and some were sliced to improve the mobility of the valve leaflet. The *Y* tendon cord was resected and an artificial tendon cord was erected using CV-4 (W. L. Gore & Associates, Inc., AZ, USA). The length of the artificial tendon cord was adjusted to a length that would ensure coaptation and ligature (Figs. 4 and 5).

**Fig. 6:**
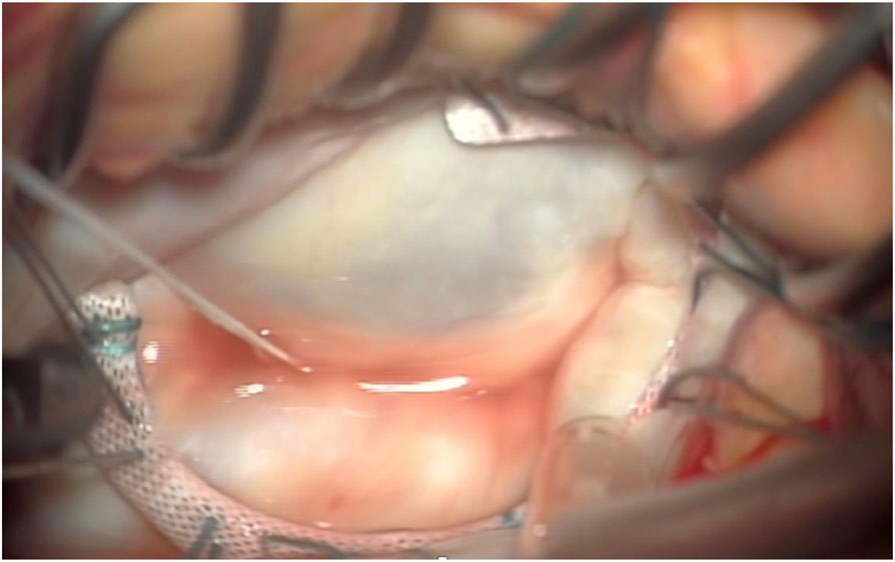
Annuloplasty using an artificial ring (Physio flex 32 mm) is performed, and a water test is performed to check for leakage and confirm MR control

We performed an annuloplasty using an artificial ring (Physio flex 32 mm, Edwards Lifesciences, Irvine, CA, USA) and water test to check for leakage and confirm MR control (Fig. 6). Because of the long duration of chronic atrial fibrillation, arrhythmia surgery was not performed, but only left atrial appendage closure was performed using an Atrial clip (50 mm). Finally, the surgery was terminated by cardiopulmonary bypass removal. The operative, cardiopulmonary bypass, and aortic cross-clamp times were 240, 124, and 78 min, respectively.

The patient’s postoperative course was uneventful, and MR was minimal on postoperative echocardiography. The patient was discharged 15 days after surgery.

## Discussion

The papillary muscles in this case were undifferentiated in both the anterior and posterior apex. The anterior apex was not treated because, as shown on the preoperative CT, there was no limitation of mobility. The undifferentiated papillary muscles on the medial and lateral sides of the posterior apex showed decreased mobility, so a partial resection and slice were performed, which successfully improved mobility.

Since the tendon cords of P2 were too short to improve mobility even if they were sliced, they were completely resected and computation was secured using artificial tendon cords.

The papillary muscles and tendon cords are thought to develop as follows: first, a horseshoe-shaped ridge, continuous with the endocardial bed, develops in the ventricle. The endocardial floor differentiates into the mitral and tricuspid valves, the horseshoe-shaped ventricular ridges change into two papillary muscles, and the papillary muscles attached to the valves gradually differentiate into tendon cords [1]. The lack of differentiation from papillary muscles to tendon cords during this process may cause the direct attachment of papillary muscles to the valve leaflets, as observed in the present case. Parachute mitral valve is a similar condition thought to be caused by the horseshoe-shaped ridge not separating into two papillary muscles but instead developing as a single papillary muscle [2]. Previously, the UDPM was considered a subtype of the parachute mitral valve; however, the aforementioned difference in mechanism has led to the distinction between those with UDPM and the parachute mitral valve.

Congenital MR due to abnormal attachment of the papillary muscles or tendon cords is relatively rare. Most reports of MR due to UDPM have involved cases in which only the anterior papillary muscle was undifferentiated [2, 3]. This present case was different in that the papillary muscle derived from the posterior papillary muscle was also undifferentiated, and both the anterior and posterior papillary muscles were undifferentiated and attached to the anterior apex. A prior study reported that of 28 cases with UDPM, 26 had undifferentiated anterior papillary muscles, and 2 had undifferentiated posterior papillary muscles [2]. No cases in which both papillary muscles were undifferentiated were observed, and this seems to be a rather rare occurrence. Although MVR is relatively safe unless complicated congenital cardiac malformations coexist, MVP is preferable, especially in younger patients. This is because MVP allows for a greater valvular area and reduces the need for long-term anticoagulation therapy, taking future physical growth into account. Owing to the congenital nature of the disease and reportedly high rate in young patients, valvuloplasty should be considered [4].

The two major causes of MR due to UDPM are thought to be reduced mobility, as only the papillary muscles are attached, and deviation due to the absence of tendon cords. In the present case, regurgitation was caused by decreased mobility. The tendon cords directly contiguous to the left ventricle, which had been identified on the preoperative cardiac CT scan, were transected, and other club-shaped papillary muscles were sliced to improve mobility. The detailed morphological abnormalities identified on cardiac CT allowed for correct treatment and intraoperative decision-making regarding the area of the mitral valve that restricts mobility.

## Conclusion

Cardiac CT enabled the preoperative diagnosis of detailed morphological abnormalities in UDPM, which helped ensure the success of mitral valvuloplasty.

## Data Availability

Not applicable.
